# Interesting and Rare Case

**Published:** 1894-04

**Authors:** E. S. Talbot

**Affiliations:** Chicago


### INTERESTING AND RARE CASE.

BY E. S. TALBOT, D.D.S., CHICAGO.

THE following interesting case is now under my care. It is so uncommon that I deem it of sufficient importance to not only place it on record, but to ask if any one has had a similar one, and also ask the cause. Mr. J. J. McG., a retired business-man, fifty-eight years of age, has lost all his bicuspids and molars upon the upper jaw. For years he has been using the incisors and cuspids for cutting and chewing his food; the result of which is, the inferior teeth have worn away, exposing and destroying the pulps of the central incisors. I made and fitted a plate upon the upper jaw, bringing the jaws to their natural position, as a result of which quite a space exists between the upper and lower incisors. For the past two weeks I have been treating the roots of the inferior central incisors with the view to filling them. Now for the mystery. A sharp edge existed upon the posterior surface of the inferior incisors. This being removed, the two teeth with dead pulps became so sensitive where they were ground away that the least touch could scarcely be borne. The pain was precisely like that of any tooth with a live nerve ground down to the dentine. After repeated applications of nitrate of silver for two weeks the pain ceased.

No pulp being present, what produced the pain?

